# Water and beverage consumption among children aged 4–13 years in France: analyses of INCA 2 (Étude Individuelle Nationale des Consommations Alimentaires 2006–2007) data

**DOI:** 10.1017/S1368980015003614

**Published:** 2016-02-16

**Authors:** Florent Vieux, Matthieu Maillot, Florence Constant, Adam Drewnowski

**Affiliations:** 1 MS-Nutrition, Marseille, France; 2 Nestlé Waters M.T., Issy-les-Moulineaux, France; 3 Center for Public Health Nutrition, University of Washington, Box 353410, Seattle, WA 98195, USA

**Keywords:** Water, Water consumption, Beverage consumption, Water intake recommendation

## Abstract

**Objective:**

To examine the consumption of plain water among children in France and compare total water intakes with guidelines issued by the European Food Safety Authority (EFSA).

**Design:**

Nationally representative data were used to assess food, beverage and water consumption by sex, age group (4–8 years, 9–13 years), income-to-poverty ratio, eating occasion and location. Beverages were classified into nine groups: water (tap or bottled), milk, 100 % fruit juice, sodas, fruit drinks, hot beverages, sports drinks and flavoured waters. Total water volume in relation to energy intake (litres/kcal) was also examined.

**Setting:**

INCA 2 study (Étude Individuelle Nationale des Consommations Alimentaires 2006–2007).

**Subjects:**

French children (*n* 835) aged 4–13 years.

**Results:**

Total water intakes were accounted for by plain water (34 %), beverages (26 %) and food moisture (40 %). Plain water could be tap (18 %) or bottled (16 %). Older children drank more plain water than did younger children and boys drank more plain water than did girls. No socio-economic gradient for plain water consumption was observed. About 90 % of children did not meet the EFSA water intake recommendations. The daily water shortfall ranged from 367 to 594 ml/d. Water-to-energy ratio was 0·75–0·77 litres/1000 kcal (4184 kJ). Children drank milk at breakfast and plain water during lunch and dinner. Caloric beverages provided 10 % of dietary energy; consumption patterns varied by eating location.

**Conclusions:**

Total water intakes among young children in France were below EFSA-recommended levels. Analyses of beverage consumption patterns by eating occasion and location can help identify ways to increase water consumption among children.

Total water requirements to meet hydration needs can be met by plain drinking-water, by water from caloric and non-caloric beverages, and by moisture from foods^(^ ^1^ ^–^ ^3^ ^)^. In general, plain water and beverages supply much more of total daily water than does food moisture^(^ ^4^ ^,^ ^5^ ^)^. Plain water and beverages supply 65–75 % of total water, while foods supply another 25–35 %, depending on age^(^ ^4^ ^,^ ^5^ ^)^.

The Dietary Reference Intake (DRI) values for total water, issued by the European Food Safety Authority (EFSA), are based in part on observed population intakes of plain drinking-water (tap and bottled), water from beverages and food moisture. Whereas the consumption of caloric beverages by children is well documented^(^ ^5^ ^,^ ^6^ ^)^, there are fewer studies on plain water consumption among nationally representative samples of children and adolescents. Further, published studies on water consumption patterns in the USA^(^ ^5^ ^,^ ^7^ ^,^ ^8^ ^)^, Mexico^(^ ^9^ ^)^, Germany^(^ ^10^ ^,^ ^11^ ^)^, Belgium^(^ ^11^ ^,^ ^12^ ^)^ and France^(^ ^11^ ^,^ ^13^ ^)^ have not always been compared with national or international recommendations and guidelines.

The EFSA-recommended values are 1600 ml/d for boys and girls aged 4–8 years; 1900 ml/d for girls and 2100 ml/d for boys aged 9–13 years^(^ ^1^ ^)^. These EFSA values may be used as goals for individual adequate intakes^(^ ^1^ ^)^. Based on water-to-energy ratio, the desirable total water intake should be in the range of 1·0–1·15 litres/1000 kcal (4184 kJ)^(^ ^1^ ^)^. EFSA has also used urine osmolality as an index of adequate hydration^(^ ^14^ ^–^ ^16^ ^)^.

In the present study, the nationally representative INCA 2 database (Étude Individuelle Nationale des Consommations Alimentaires 2006–2007)^(^ ^17^ ^,^ ^18^ ^)^ was used to assess total water consumption among French children aged 4–13 years. The INCA 2 study is the flagship national dietary survey, conducted by the French National Agency for Food Safety (ANSES). The INCA 2 data are the most recent publicly available for France and are used to inform national food and nutrition policy, most notably the National Program for Nutrition and Health^(^ ^19^ ^)^.

The present goal was to compare how close French children came to meeting the EFSA Dietary Reference Values for water. Additional analyses estimated total water sources (plain water, beverages and foods) and the water-to-energy ratio (litres/kcal) in relation to desirable norms. Analyses were conducted by sex and age group and by socio-economic status, eating occasion and location. The contributions of beverages and foods to total energy intake were also examined.

## Methods

### Dietary intake databases

The INCA 2 study was conducted by the French National Agency for Food Security and Safety (AFSSA) between December 2005 and April 2007^(^ ^17^ ^,^ ^18^ ^)^. INCA 2 provides data from a nationally representative sample of 4119 persons aged 3–79 years. Dietary intake assessment was based on 7 d food records for all foods and beverages consumed, including plain water^(^ ^20^ ^)^. Participants were asked to describe all of the foods and beverages consumed and estimate the amounts consumed, aided by a photographic atlas of portion sizes and a list of household measures. For children aged 3–10 years, food records were completed by responsible parents or caregivers. For children aged >10 years, the child was the primary source of dietary recall information, but could be assisted by an adult who had knowledge of the child’s diet. Separate eating occasions were defined as breakfast, morning snack, lunch, afternoon snack, dinner and evening snack. Eating locations were provided as well.

### Age, sex and socio-economic strata

Separate analyses were conducted for boys and girls. The age groups were 4–8 years and 9–13 years. To calculate income-to-poverty ratio (IPR), reported household incomes were first divided by an adjusted number of persons in the household to arrive at income per person. The French practice is to assign a weight of 1 to the first adult, 0·5 to other persons aged >14 years and 0·3 to persons aged <14 years. The IPR cut-off points were defined as <1·0, 1·0–1·99, 2·0–3·49 and ≥3·5, following previous studies^(^ ^4^ ^,^ ^5^ ^)^. The poverty threshold was defined as 60 % of median income in 2007 or 908 Euros per month. Previously published methods^(^ ^21^ ^)^ were used to address missing income data. The Kohonen algorithm imputed missing income data based on age, sex, socio-occupational status, level of education and marital status, as well as standard-of-living variables (homeowner or not, home equipment)^(^ ^21^ ^)^.

### Plain water and beverage consumption

Plain water and beverages were classified into nine categories: (i) tap water; (ii) bottled water; (iii) milks (including flavoured); (iv) sodas (regular and diet); (v) 100 % fruit juices; (vi) hot beverages (coffee and tea); (vii) fruit drinks; (viii) sports drinks; and (ix) flavoured waters. The INCA 2 food records for each respondent provided information on the amount in grams of each food and beverage consumed^(^ ^17^ ^,^ ^18^ ^,^ ^20^ ^)^. The water content of beverages and the moisture content of foods were established using the CIQUAL (Centre d’Information sur la Qualité des Aliments) 2013 database developed by the ANSES. Food and beverage amounts were converted to energy (kcal; 1 kcal=4·184 kJ) using the CIQUAL database and standard procedures. The comparisons with the EFSA total water guidelines presented herein were for water content from different sources, including plain water, beverages and foods, calculated in ml/d. By contrast, analyses of water and beverage consumption by eating occasion and eating location were based on the mean total weight of plain water and water from beverages calculated in g/d.

### Statistical analyses

Analyses evaluated the survey-weighted, mean 7 d intake of total water overall and by age group, sex and IPR. The consumption of plain water, tap and bottled, was evaluated separately for the entire population and for subgroups of interest. The contribution of other beverages and food moisture to total water intake was also examined. All analyses by sex, age group and IPR were based on ANOVA with *post hoc* comparisons between means using Bonferroni correction. Tests of percentages of children failing to meet EFSA recommendations were based on the non-parametric *χ* ^2^ test. The estimated percentage of children failing to meet the DRI represents the lower bound of the number of children who meet the recommended intake level since the mean of 7 d water intake may not represent the habitual intake of an individual. All analyses accounted for the complex survey design of INCA 2 and reflect the behaviours of the French child population from December 2005 to April 2007. The tests were conducted using the statistical software package SAS version 9·4 and the SURVEYREG, SURVEYMEANS and SURVEYFREQ procedures.

## Results

### Plain water consumption

Table 1 shows the consumption of plain water in ml/d by age, sex and sociodemographic group. On average, children aged 4–13 years drank a total of 453 ml water/d as a beverage. Younger children (4–8 years) drank 408 ml water/d whereas older children (9–13 years) drank 499 ml/d. Boys drank more water than did girls (489 *v*. 412 ml/d).

**Table 1:** Consumption of plain water (total, tap and bottled, in ml/d) by age group, sex and sociodemographic group; French children aged 4–13 years (*n* 835), INCA 2 (Étude Individuelle Nationale des Consommations Alimentaires 2006–2007)

		Total plain water (mld)		Tap water (ml/d)		Bottled water (ml/d)	
	*n*	Mean	95 % CI	Mean	95 % CI	Mean	95 % CI
All children	835	453·0	431·6, 474·4	242·5	219·2, 265·9	210·5	190·6, 230·3
Age group							
4–8 years	355	407·6	373·0, 442·2	207·8	174·5, 241·2	199·8	172·5, 227·0
9–13 years	480	498·6	461·2, 535·9	277·4	243·2, 311·5	221·2	192·4, 250·0
*P* value		**<0**·**0001**		**<0**·**001**		0·2664	
Sex							
Boys	400	489·0	448·1, 529·9	267·3	230·4, 304·3	221·7	189·1, 254·2
Girls	435	411·9	383·3, 440·5	214·2	184·6, 243·8	197·7	174·5, 220·9
*P* value		**<0**·**001**		**<0**·**01**		0·2348	
Age × sex groups							
Boys 4–8 years	172	434·6	383·5, 485·7	239·0	190·9, 287·0	195·6	150·2, 241·0
Boys 9–13 years	228	543·3	489·5, 597·2	295·7	247·4, 344·0	247·7	205·0, 290·4
Girls 4–8 years	183	377·0	344·6, 409·5	172·5	139·2, 205·9	204·5	174·1, 234·8
Girls 9–13 years	252	447·2	406·7, 487·7	256·4	216·3, 296·5	190·8	159·9, 221·7
*P* value		**<0**·**0001**		**<0**·**0001**		0·1542	
Family income-to-poverty ratio*							
<1	356	433·3	397·3, 469·3	246·6	208·1, 285·0	186·7	157·2, 216·3
1–1·99	327	479·3	429·3, 529·3	250·4	213·2, 287·6	228·9	186·4, 271·5
≥2	152	438·1	399·0, 477·2	214·0	172·6, 255·3	224·2	182·1, 266·2
*P* value		0·2087		0·3512		0·1840	

*P* value is for ANOVA with *post hoc* Bonferroni-adjusted comparisons; significant *P* values are indicated in bold font.

* ‘<1’ means below the poverty threshold; ‘≥2’ means more than two times higher than the poverty threshold.

The intake of tap *v*. bottled water in ml/d is also shown in Table 1. Overall, children aged 4–13 years consumed 242 ml tap water (53 %) and 210 ml bottled water (47 %) daily. Tap water consumption was higher for older children and for boys. For bottled water, there were no significant differences by sex or age group. Children living in lower-income households were as likely to consume plain water as were children living in higher-income households.

### Water intake from plain water, beverages and foods

Table 2 summarizes the principal sources of total daily water intake (ml/d) for the total sample and for subgroups of interest. Total water intake from plain water, beverages and foods was estimated at 1324 ml/d. Tap and bottled water together contributed 453 ml/d or 34 % of total water (tap 18 % and bottled 16 %), whereas caloric and non-caloric beverages contributed a further 348 ml/d (26 %). Plain water and beverages together contributed 801 ml (60 %) of total water daily, with a further 524 ml/d (40 %) provided by food moisture.

**Table 2:** Intakes (ml/d) of total water, plain water and water from beverages and foods* by age group, sex and sociodemographic group; French children aged 4–13 years (*n* 835), INCA 2 (Étude Individuelle Nationale des Consommations Alimentaires 2006–2007)

		Total water intake (ml/d)		Plain water (ml/d)		Water from beverages (ml/d)		Water from solid foods (ml/d)	
	*n*	Mean	95 % CI	Mean	95 % CI	Mean	95 % CI	Mean	95 % CI
All children	835	1324	1295, 1353	452·7	431·3, 474·1	347·8	334·6, 361·0	523·8	512·5, 535·1
Age group									
4–8 years	355	1233	1193, 1273	407·4	372·8, 441·9	333·3	314·3, 352·3	492·3	474·9, 509·7
9–13 years	480	1416	1368, 1464	498·2	460·9, 535·6	362·4	342·2, 382·6	555·5	540·9, 570·0
*P* value		**<0**·**0001**		**<0**·**0001**		**<0**·**05**		**<0**·**0001**	
Sex									
Boys	400	1393	1345, 1441	488·7	447·8, 529·6	363·8	344·9, 382·6	540·7	520·6, 560·8
Girls	435	1246	1207, 1285	411·6	383·1, 440·2	329·6	311·2, 348·1	504·6	490·1, 519·1
*P* value		**<0**·**0001**		**0**·**0005**		**<0**·**05**		**<0**·**01**	
Age × sex groups									
Boys 4–8 years	172	1280	1221, 1339	434·3	383·4, 485·2	344·4	317·1, 371·7	501·2	476·0, 526·3
Boys 9–13 years	228	1506	1443, 1570	543·0	489·0, 597·0	383·1	356·0, 410·1	580·2	558·8, 601·5
Girls 4–8 years	183	1180	1131, 1228	376·8	344·4, 409·3	320·7	294·2, 347·2	482·3	459·0, 505·6
Girls 9–13 years	252	1313	1249, 1377	446·9	406·4, 487·3	338·6	310·4, 366·9	527·2	501·6, 552·7
*P* value		**<0**·**0001**		**<0**·**0001**		**<0**·**01**		**<0**·**0001**	
Income-to-poverty ratio†									
<1	356	1315	1267, 1363	433·0	397·0, 469·0	367·7	345·3, 390·0	514·4	494·9, 533·9
1–1·99	327	1332	1274, 1390	479·0	429·0, 529·0	330·6	309·3, 351·9	522·7	504·1, 541·4
≥2	152	1328	1263, 1392	437·9	398·8, 476·9	340·5	313·0, 368·0	549·3	525·1, 573·6
*P* value		0·9066		0·2089		0·0668		0·0905	

*P* value is for ANOVA with *post hoc* Bonferroni-adjusted comparisons; significant *P* values are indicated in bold font.

* Water content of all beverages and foods was calculated using the CIQUAL (Centre d’Information sur la Qualité des Aliments) 2013 database.

† ‘<1’ means below the poverty threshold; ‘≥2’ means more than two times higher than the poverty threshold.

Analyses of total water intake by age group showed that plain water contributed 33 % of water intake in the 4–8 years age group and 35 % in the 9–13 years age group. Plain water accounted for 35 % of total water intake among boys and 33 % among girls. Total water intake was significantly higher for the oldest children and boys. There were no differences in total water intake by income.

**Fig. 1:**
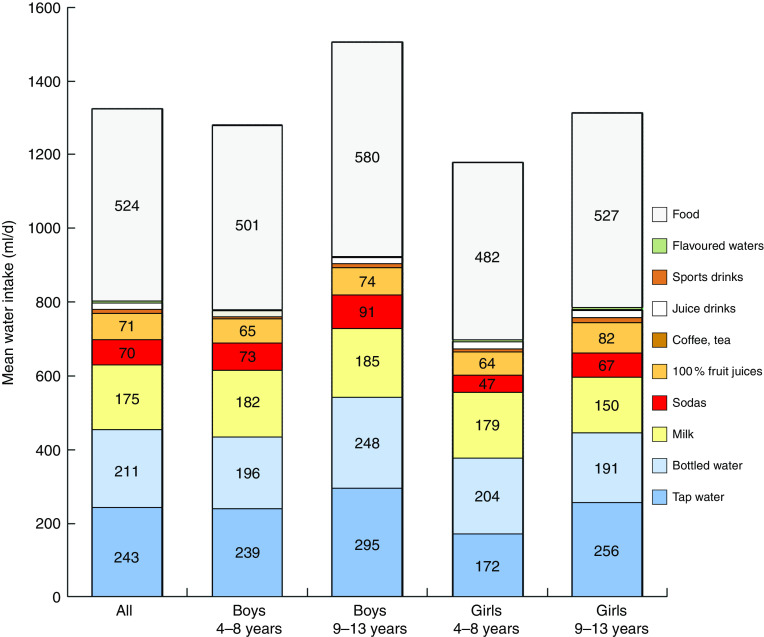
(colour online) Mean water intake (ml/d) from different sources for the total sample (all) and by age group and sex; French children aged 4–13 years (*n* 835), INCA 2 (Étude Individuelle Nationale des Consommations Alimentaires 2006–2007)

Figure 1 shows total water intake (ml/d) from all sources by age group and sex. The beverages were separated into categories. Milk contributed 175 ml or 13·2 % of total daily water. Soda accounted for 70 ml or 5·3 %; 100 % fruit juices for 71 ml or 5·5 %; and fruit drinks for 17 ml or 1·2 %. Coffee and tea, juice-based beverages, sports drinks and flavoured water contributed very modest amounts (<1 %). In general, older children consumed more total water than did younger children (*P*<0·0001); however, no significant differences across age group by beverage type were observed, except for tap water (*P*=0·0007). Water intake from food moisture also varied by age group (*P*<0·0001).

### Total water intakes compared with recommendations

**Fig. 2:**
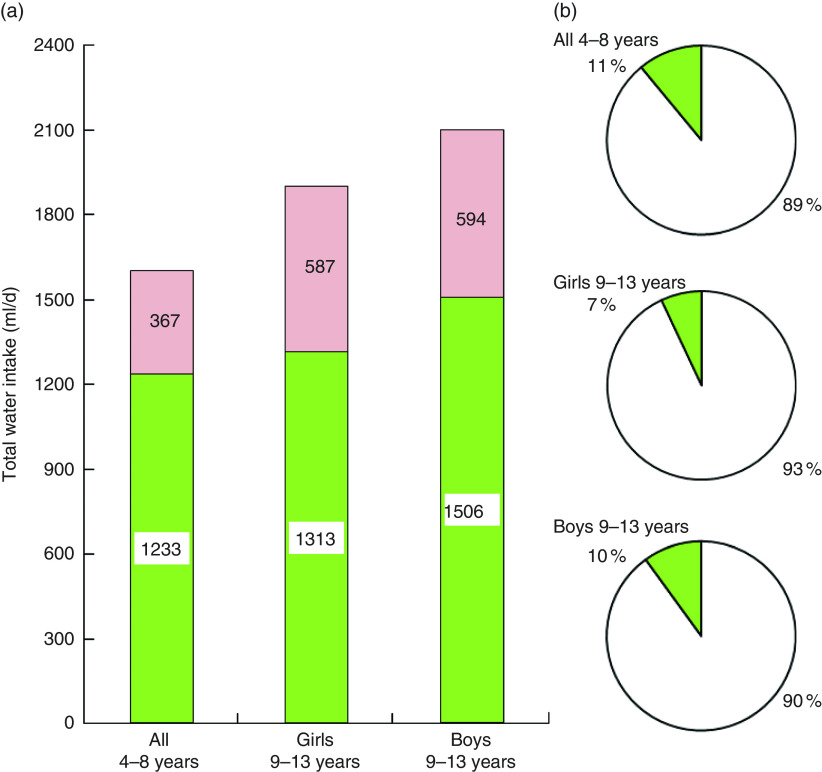
(colour online) (a) Total water intake (ml/d) by age group and sex (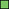) in relation to the European Food Safety Authority (EFSA) recommendations; the amount of water shortage is also indicated (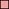). (b) The proportion of children (%) by age group and sex who did (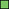) or did not (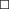) meet EFSA recommendations. French children aged 4–13 years (*n* 835), INCA 2 (Étude Individuelle Nationale des Consommations Alimentaires 2006–2007)

Figure 2 shows that, on average, no group of children met the EFSA recommendations. The shortfall in water consumption relative to EFSA values ranged from 367 ml/d (4–8 years) to 594 ml/d (boys aged 9–13 years). Depending on sex and age group, only 7–11 % of children met the EFSA recommendations. Younger children were not stratified by sex because the EFSA recommendation for that age group is the same for boys and girls.

The desirable water-to-energy ratio based on EFSA norms is of the order of ≥1000 ml/1000 kcal (4184 kJ)^(^ ^1^ ^)^. The observed ratio for children aged 4–13 years was 761 ml/1000 kcal, or 0·76. For boys the ratio was 0·75 and for girls it was 0·77. These values fell far short of EFSA recommendations and were much lower than the values observed for the same age group in the USA.

### Plain water and beverage consumption by eating occasion and location

**Fig. 3:**
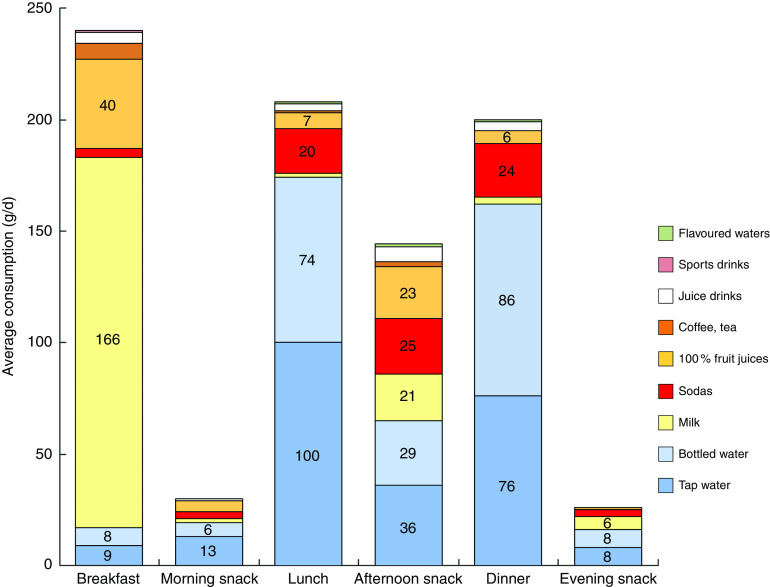
(colour online) Average consumption (g/d) of water and beverages by eating occasion; French children aged 4–13 years (*n* 835), INCA 2 (Étude Individuelle Nationale des Consommations Alimentaires 2006–2007)

Figure 3 shows the consumption (in g/d) of beverages, including tap and bottled water, by eating occasion. Milk accounted for 69·7 % of all beverages consumed at breakfast. Tap water accounted for 48·3 % of beverages consumed at lunch, whereas bottled water contributed another 35·6 %. Tap water accounted for 38·1 % of beverages consumed at dinner and bottled water accounted for 42·8 %. Tap and bottled waters were less likely to be consumed during the afternoon snack. Soda consumption in this group of young French children was relatively low.

**Fig. 4:**
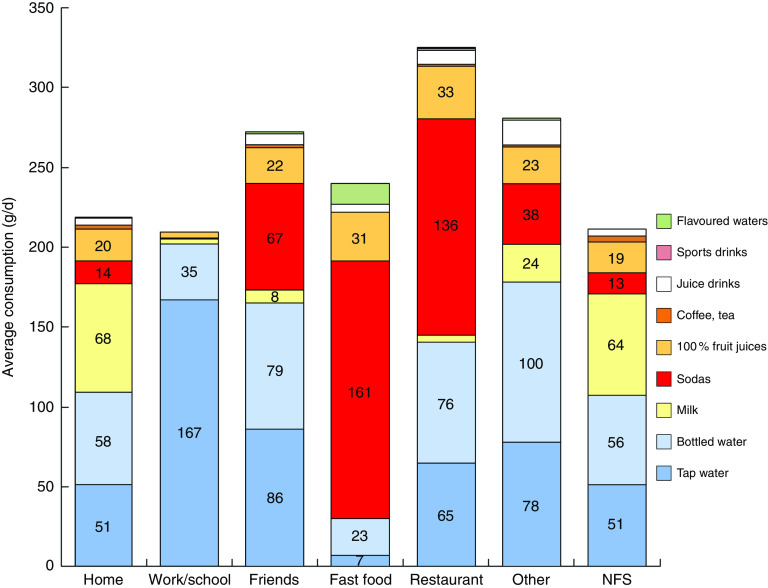
(colour online) Average consumption (g/d) of water and beverages by eating location (NFS, not further specified); French children aged 4–13 years (*n* 835), INCA 2 (Étude Individuelle Nationale des Consommations Alimentaires 2006–2007)

Figure 4 shows average amounts of water and beverages (in g/d) by eating location. Not every child consumed water or beverages at every location, so the numbers of children per location differed widely. Eating locations were defined as home (*n* 831), school canteen (*n* 445), friend’s house (*n* 241), fast-food restaurant (*n* 94), restaurant (*n* 11), other location (*n* 304) and NFS (not further specified; *n* 434). It can be seen that water and milk were the main beverages consumed at home, whereas water (tap and bottled) was the main beverage consumed in school canteens and at friends’ homes. The consumption of sodas by children in France was highest in restaurants and especially at fast-food restaurants.

### Dietary energy from beverages and foods

Table 3 shows the relative contributions of caloric beverages and foods to total energy intake as a function of age group and sex. The data are presented as kilocalories and as percentages. It can be seen that beverages contributed about 11 % of energy to the total diet, with the bulk of energy provided by solid foods. Younger children derived a greater proportion of energy from beverages than did older children (11·5 % *v*. 10·1 %).

**Table 3:** Contribution (as kcal* and %) of beverages and foods to total daily energy intake by age group and sex; French children aged 4–13 years (*n* 835), INCA 2 (Étude Individuelle Nationale des Consommations Alimentaires 2006–2007)

	Beverages (kcal)		Foods (kcal)		Beverages (%)		Foods (%)	
	Mean	95 % CI	Mean	95 % CI	Mean	95 % CI	Mean	95 % CI
All children	189	181, 196	1580	1548, 1611	10·8	10·4, 11·2	89·2	89·1, 90·4
Age group								
4–8 years	188	177, 199	1461	1417, 1505	11·5	10·9, 12·1	88·5	87·8, 89·1
9–13 years	190	179, 201	1699	1656, 1741	10·1	9·6, 10·6	89·9	89·4, 90·4
*P* value	0·7815		**<0**·**0001**		**0**·**0007**		**0**·**0007**	
Sex								
Boys	202	191, 212	1670	1621, 1718	10·9	10·3, 11·5	89·1	88·5, 89·7
Girls	175	165, 184	1477	1439, 1514	10·7	10·1, 11·2	89·3	88·7, 89·9
*P* value	**0**·**0002**		**<0**·**0001**		0·5467		0·5467	
Age × sex groups								
Boys 4–8 years	197	182, 213	1531	1466, 1595	11·6	10·6, 12·3	88·4	87·5, 89·3
Boys 9–13 years	206	191, 221	1808	1751, 1865	10·2	9·5, 10·9	89·8	89·1, 90·4
Girls 4–8 years	177	162, 192	1382	1333, 1431	11·5	10·6, 12·3	88·5	87·7, 89·4
Girls 9–13 years	172	157, 187	1573	1518, 1628	9·9	9·1, 10·7	90·1	89·3, 90·9
*P* value	**0**·**0027** ^**a**^		**<0**·**0001** ^**b**^		**0**·**0093** ^**a**^		**0**·**0093** ^**a**^	

*P* value is for ANOVA with *post hoc* Bonferroni-adjusted comparisons; significant *P* values are indicated in bold font.

a No significant differences between means.

b Difference in means for Girls 9–13 years *v*. Boys 4–8 years not significant.

* 1 kcal=4·184 kJ.

## Discussion

Analyses based on a representative sample of 4- to 13-year-old children in France, using the most recent INCA 2 database^(^ ^17^ ^,^ ^18^ ^,^ ^20^ ^)^, estimated total water consumption at 1324 ml/d and plain water consumption at 453 ml/d. Beverage consumption was 348 ml/d. About 90 % of boys and girls had a total water intake below EFSA recommendations. For boys and girls aged 9–13 years, total water intake was about 0·6 litres short of the DRI. The second criterion of adequate hydration was in the range of 0·76 litres water/1000 kcal (4184 kJ) as opposed to the desirable 1·0 litres/1000 kcal.

In studies based on data from the US National Health and Nutrition Examination Survey (NHANES)^(^ ^5^ ^)^, total water intake for the same 4–13 years age group was estimated at 1580 ml/d and plain water consumption at 431 ml/d. Beverage consumption was estimated at 704 ml/d, including both milk and caloric and non-caloric beverages^(^ ^5^ ^)^. Even so, no group of US children came close to satisfying the DRI for water, using values issued by the US Institute of Medicine. At least 75 % of US children aged 4–8 years, 87 % of girls aged 9–13 years and 85 % of boys aged 9–13 years did not meet the DRI for total water intake^(^ ^5^ ^)^. The water-to-energy ratio was 0·85–0·95 litres/1000 kcal (4184 kJ). For this age group, bottled water contributed 40 % to plain water intake in the USA and 46 % in France. Whereas the consumption of plain water appeared to be similar in the USA and France (453 *v*. 431 ml/d), US children consumed significantly more other beverages, including sweetened beverages and milk (704 *v*. 348 ml/d).

Comparable to the USA, a recent study based on data from the Mexican National Health and Nutrition Survey 2012^(^ ^9^ ^)^ for children and adolescents aged 1–18 years estimated total water consumption at 1614 ml/d and plain water at 427 ml/d. Beverage consumption was estimated at 630 ml/d, with different proportions from milk, *agua fresca* (fruit water) and soda depending on age. A majority of Mexican children failed to meet the DRI for water.

The INCA 2 national dietary survey, conducted in 2006–2007 by ANSES, continues to inform the national food and nutrition policy. The present findings, based on the most recent INCA database, show that French children consumed mostly milk at breakfast and plain water at lunch and at dinner meals. The beverage consumed in school canteens was almost exclusively plain water. Promoting water consumption in schools is the goal of French national policies and guidelines^(^ ^19^ ^)^. Sodas were more likely to be consumed in full-service and fast-food restaurants. On the average, about 10 % of daily energy in the French children’s diets came from caloric beverages, including milk. Interestingly, and in contrast to consumption data from the USA and Mexico^(^ ^4^ ^,^ ^5^ ^,^ ^9^ ^)^, plain water consumption among French children was not associated with higher household incomes.

Studies of beverage consumption by children have tended to focus on caloric beverages such as milk^(^ ^22^ ^)^, fruit juices^(^ ^23^ ^)^ and sweetened beverages^(^ ^24^ ^,^ ^25^ ^)^. By and large, the focus was on the amount of dietary energy provided in liquid form. In contrast, fewer studies have focused on the patterns of water consumption by age group, sex, socio-economic status or drinking occasion. Even fewer studies have compared observed intakes with the existing recommendations. Our age cut-off points were deliberately similar to those used in publicly available recommendations and guidelines. For example, the Scientific Opinion on Dietary Reference Values for Water published by EFSA^(^ ^1^ ^)^ noted that adolescents aged ≥14 years were considered as adults with respect to adequate water intake. Since the present goal was to compare observed fluid consumption patterns with public health guidelines, we needed to follow EFSA-imposed age cut-off points exactly. Although different age cut-off points have been used in published literature, they are not always directly translatable to public policy guidelines.

One previous study from France^(^ ^13^ ^)^ examined fluid intake from beverages in a sample of healthy French children, adolescents and adults. However, water consumption was not the main focus. Beverages were divided into five categories (water, hot drinks, juice, sodas and dairy drinks), plus alcohol. The age cut-off points were 6–11 years for children and 12–19 years for adolescents. Dietary data came from the CCAF survey (Comportement et Consommations Alimentaires en France study), a stratified population sample, recruited using a quota system and conducted in 2002–2003^(^ ^13^ ^)^. By contrast, beverages in the present paper were classified into nine categories (tap water, bottled water, milks (including flavoured), sodas (regular and diet), 100 % fruit juices, hot beverages (coffee and tea), fruit drinks, sports drinks and flavoured waters). Children were stratified by age, sex and socio-economic status. Plain water was split into bottled and tap water. The present paper provides a more recent and a far more comprehensive picture of fluid consumption by children in France and can be linked directly to initiatives in public health policy.

A comparison of water and beverage consumption patterns by 9- to 13-year-olds showed that boys in Belgium^(^ ^12^ ^)^ consumed 920 ml water and beverages daily whereas boys in France^(^ ^13^ ^)^ consumed 926 ml/d. For girls aged 9–13 years water and beverages supplied 836 ml/d in Belgium and 785 ml/d in France. Both studies concluded that the observed consumption was below recommended values.

The present focus on plain water is justified by important policy implications. The French National Plan for Nutrition and Health (PNNS) recommends that plain drinking-water be the principal beverage consumed both during and between meals^(^ ^19^ ^,^ ^26^ ^,^ ^27^ ^)^. The PNNS also recommends that schools make free drinking-water available to students when meals are served, installing fresh water fountains to provide students with easy access to a non-caloric beverage at no charge^(^ ^26^ ^–^ ^28^ ^)^. Similarly, GEMRCN (Groupe d’Etude des Marchés Restauration Collective et Nutrition), the French national administrative authority with responsibility for school canteens, has issued guidelines to improve the quality of school meals^(^ ^29^ ^)^. Plain drinking-water is the only permitted beverage. Whereas unsweetened low-fat milk can be permitted, the consumption of sodas is discouraged. The ESPGHAN (European Society for Paediatric Gastroenterology Hepatology and Nutrition) Committee on Nutrition^(^ ^30^ ^)^ considers that plain water should be promoted as the main source of fluids for children. Drinking plain water instead of caloric beverages may also help reduce dietary energy density and help in the management of body weight^(^ ^31^ ^–^ ^34^ ^)^. Similar initiatives to increase the availability and consumption of plain drinking-water in schools have also been explored in the USA^(^ ^35^ ^)^. Studies on substitution of sweetened beverages with alternatives were recently reviewed^(^ ^36^ ^)^.

The present study allows for the first direct comparison of beverage consumption patterns by children aged 4–13 years in France and the USA. That per capita consumption of bottled water is higher in France than in the USA is well established^(^ ^37^ ^)^. The consumption of sodas by children has been reported to be lower in France than in the USA^(^ ^5^ ^,^ ^13^ ^)^. The present analyses of the INCA 2 database^(^ ^38^ ^)^ show that compared with US children, the consumption of plain water by French children was much higher and the consumption of both milk and soda was considerably lower. A much higher proportion of dietary water came from moisture in foods, suggesting a higher consumption of low-energy-density foods such as vegetables and fruits. Nevertheless, in both studies children are not meeting the daily needs for water as recommended respectively by EFSA and the Institute of Medicine.

The present analyses had limitations. First, the INCA 2 data, based on self-report, are subject to random inaccuracies and systematic reporting biases. The proxy recall for younger children may be an additional source of bias. The 7 d food records impose a burden on respondents and data quality can be variable. Validation data have been published before^(^ ^39^ ^)^. Most important, the INCA 2 data were collected in 2006–2007. Given rapid shifts in beverage consumption patterns, especially by children and adolescents, the data are in danger of becoming obsolete. For example, recent data from the USA showed a plunge in added sugars consumption among children and adolescents aged 2–19 years^(^ ^32^ ^)^. While the INCA 3 study is already underway, the INCA 2 database remains the standard source of information about dietary intakes in France and is comparable in scope and importance to NHANES in the USA. The present analyses represent one of the first explorations of the consumption of drinking-water by children in France.

Comparisons of beverage consumption patterns in France with those of same-age children in the USA can serve to inform public health professionals about the importance of improving the quality of children’s drinking habits. In addition, even if the French children consumed relatively more plain water than did children in the USA, they are not meeting the daily needs for water as recommended by EFSA. So it would be relevant to educate parents and caregivers for making sure that children drink the adequate amount of water on a daily basis.
